# A rare case of odontogenic myxofibroma of the maxilla in a young patient: a challenging diagnostic and differential diagnosis

**DOI:** 10.21142/2523-2754-1403-2026-307

**Published:** 2026-07-10

**Authors:** Prisilla Mutiara Djehan Pattisahusiwa, Raden Hari Triwijaya, Roberto Hutapea, Wendy Pelupessy, Elisabeth Indria Sari, Nadhia S Latuamury, Fiki Muhammad Ridho

**Affiliations:** 1 Department of Oral and Maxillofacial Surgery, Faculty of Medicine, Universitas Pattimura. Ambon, Indonesia. wied.haart@gmail.com Department of Oral and Maxillofacial Surgery Faculty of Medicine Universitas Pattimura Ambon Indonesia wied.haart@gmail.com; 2 Department of Oral and Maxillofacial Surgery, Buru Regency Hospital. Namlea, Indonesia. mutiaradjehan@gmail.com Department of Oral and Maxillofacial Surgery Buru Regency Hospital Namlea Indonesia mutiaradjehan@gmail.com; 3 Department of Oral and Maxillofacial Surgery, Dr. Johannes Leimena Hospital. Ambon, Indonesia. robertohutapea2308@gmail.com Department of Oral and Maxillofacial Surgery Dr. Johannes Leimena Hospital Ambon Indonesia robertohutapea2308@gmail.com; 4 Department of Dental Public Health, Faculty of Medicine, Universitas Pattimura. Ambon, Indonesia. drgwendypelupessy@gmail.com Department of Dental Public Health Faculty of Medicine Universitas Pattimura Ambon Indonesia drgwendypelupessy@gmail.com; 5 Department of Anatomical Pathology, Dr. Johannes Leimena Hospital. Ambon, Indonesia. elisabethyehudahutapea@gmail.com Department of Anatomical Pathology Dr. Johannes Leimena Hospital Ambon Indonesia elisabethyehudahutapea@gmail.com; 6 General Dentist, Puskesmas Tehoru. Maluku Tengah, Indonesia. latuamurynadhia@gmail.com General Dentist Puskesmas Tehoru Maluku Tengah Indonesia latuamurynadhia@gmail.com; 7 Department of Dental Medicine, Faculty of Dental Medicine, Universitas Airlangga. Surabaya, Indonesia. fiki.muhammad.ridho-2020@fkg.unair.ac.id Department of Dental Medicine Faculty of Dental Medicine Universitas Airlangga Surabaya Indonesia fiki.muhammad.ridho-2020@fkg.unair.ac.id

**Keywords:** maxilla, myxofibroma, odontogenic tumor, oral surgery, maxilar, mixofibroma, tumor odontogénico, cirugía oral

## Abstract

Odontogenic myxofibroma is a benign but locally aggressive, slow-growing, expansile mesenchymal tumor that is more commonly observed in women and primarily affects the posterior mandible, with rare involvement of the maxilla. This tumor accounts for approximately 3-6% of odontogenic tumors. This article describes a rare case of a 17-year-old male presenting with a two-year history of an enlargement on the right side of the face, affecting the buccal and palatal areas of the posterior maxilla. Clinically, the lesion exhibited a hard consistency with normal coloration of the surrounding tissues, was non-ulcerated and non-tender on palpation, and showed no signs of bleeding. The tumor was completely removed, and the remaining bone was stabilized using a miniplate. At the 16-month follow-up evaluation, no clinical or radiographic evidence of tumor recurrence was observed. This case highlights the diagnostic challenges associated with maxillary odontogenic myxofibroma due to its nonspecific clinical and radiographic features and underscores the importance of histopathological confirmation for accurate diagnosis and appropriate management.

## RESUMEN

El mixofibroma odontogénico es un tumor mesenquimal benigno, pero localmente agresivo, de crecimiento lento y expansivo, que se observa con mayor frecuencia en mujeres y afecta principalmente la mandíbula posterior, con rara afectación del maxilar. Este tumor representa aproximadamente el 3-6% de los tumores odontogénicos. Este artículo describe un caso poco frecuente de un varón de 17 años que presentó una historia de dos años de aumento de volumen en el lado derecho de la cara, que afectaba las áreas bucal y palatina del maxilar posterior. Clínicamente, la lesión presentó una consistencia dura con coloración normal de los tejidos circundantes, no estaba ulcerada ni era dolorosa a la palpación, y tampoco mostró signos de sangrado. El tumor fue completamente removido y el hueso remanente se estabilizó mediante el uso de una miniplaca. En la evaluación de seguimiento a los 16 meses, no se observó evidencia clínica ni radiográfica de recurrencia tumoral. Este caso pone de manifiesto los desafíos diagnósticos asociados con el mixofibroma odontogénico del maxilar debido a sus características clínicas y radiográficas inespecíficas, así como subraya la importancia de la confirmación histopatológica para un diagnóstico preciso y un manejo adecuado.

## INTRODUCTION

Odontogenic myxofibroma is a relatively rare tumor derived from benign mesenchymal spindle cells (intraosseous) and is considered a variant of odontogenic myxoma, which has been reported to account for approximately 3-6% of all odontogenic tumors ^1^. According to the World Health Organization (WHO), odontogenic myxofibroma is identical to odontogenic myxoma. The estimated annual incidence of odontogenic myxofibroma is approximately 0.07 new cases per million population ^2^. Clinically, it occurs in individuals in the second to fourth decade of life, with a higher prevalence in women and is rarely found in children and the elderly. It tends to occur in the mandible, especially in the posterior region with a high recurrence rate ^3^.

In general clinical practice, the absence of symptoms is typically observed; however, as the tumor advances, it may lead to facial asymmetry due to a gradual increase in swelling accompanied by the expansion of the cortical plate and malocclusion ^4^. This tumor is characterized by the presence of spindle-shaped cells, is proliferative in nature, and represents an accumulation of mucoid ground substance that exhibits a higher collagen content in comparison to myxoma. Myxofibroma, odontogenic fibroma, and myxoma are histogenetically regarded as part of a continuum of benign mesenchymal tumors that display heterogeneity yet exhibit distinct behavioral characteristics ^5^^,^^6^

Radiographically, myxofibroma presents as a unilocular or multilocular radiolucent lesion that usually has a well-defined border. Descriptive patterns such as "honeycomb," "soap-bubble," "opaque-glass," and "tennis racket string" appearances have been reported in the literature. In addition, root resorption is uncommon while tooth migration is relatively common ^4^.

Given the nonspecific clinical and radiographic characteristics of odontogenic myxofibroma, establishing an accurate diagnosis requires a multifaceted approach that integrates clinical findings, radiographic imaging, and histopathological evaluation ^2^. Although immunohistochemical analysis may be used as an adjunctive tool in selected cases, it is not considered a definitive diagnostic modality for this entity ^7^. This article presents a rare case of odontogenic myxofibroma of the maxilla in a young patient, highlighting the diagnostic challenges and providing considerations regarding the differential diagnosis.

## CASE REPORT

**Figure 1:**
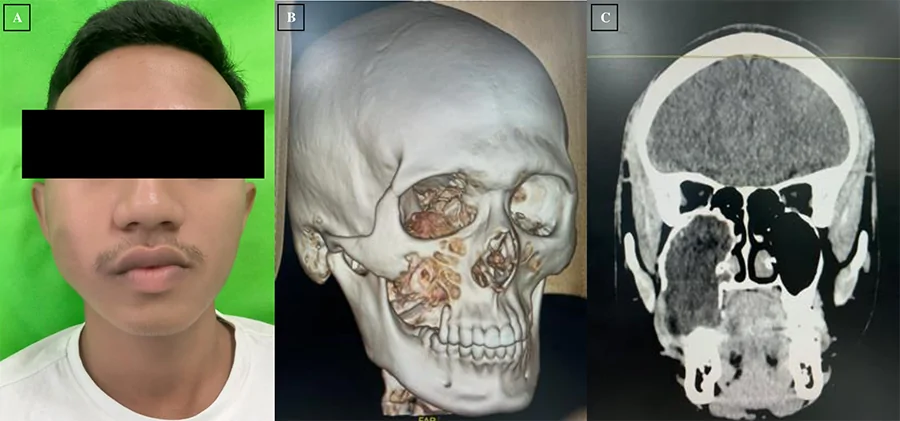
(A) Extraoral examination showing facial asymmetry; (B-C) Three-dimensional coronal CT scan view.

A 17-year-old male presented with an enlargement on the right side of his face (Figure 1A) that had been present for two years, affecting the buccal and palatal areas of the maxillary posterior teeth, measuring approximately 6 x 3 cm. The consistency of the tumor was hard with normal coloration, not-ulcerated, no-tender on palpation, and no bleeding. Mobility of the second premolar was noted. The patient denied any history of trauma or systemic disease.

Craniofacial computerized tomography (CT) scan showed a slight hyperdense lesion anterior to the right first molar extending to the premolar region. The lesion appeared to extend superiorly, destroying the medial side and medial wall of the right maxillary sinus, filling the right maxillary sinus, and destroying the medial wall of the right maxillary sinus into the nasal cavity. Inferiorly, the lesion extended to the cutis and subcutis of the maxilla region. The imaging findings raised suspicion of an aggressive lesion, including possible malignant lesion (Figure 1B-C). Based on the initial clinical assessment and subsequent CT scan findings indicating an expansive intraosseous lesion in the maxilla, diagnoses of ossifying fibroma, odontogenic myxofibroma, and fibrous dysplasia were considered.

**Figure 2:**
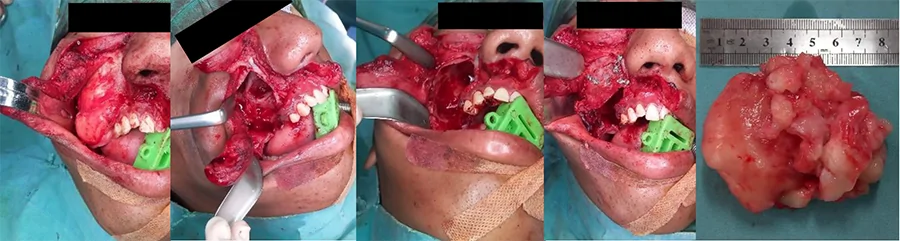
Intraoperative tumor removal (enucleation).

The procedure was performed in the operating room, with a plan to enucleate the tumor. Tumor access was done using the Weber Ferguson modification approach combined with a maxillary vestibular incision. The incision line was drawn through the vermilion border, along the philtrum of the lip, extending around the base of the nose and along the nasofacial groove. It then extended infraorbitally 3-4 mm below the cilium to the lateral canthus. Intraoperatively, a firm, elastic mass was identified, with a boundary between the intraoral and the tumor infiltrating the maxillary antrum. The tumor was completely removed, and the remaining bone plate was stabilized using a miniplate (Figure 2).

**Figure 3:**
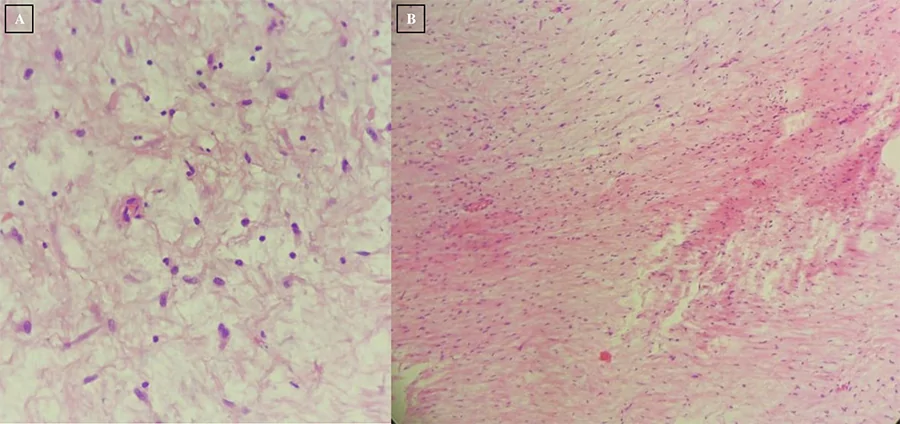
(A) Photomicrograph (x10) showing myxoid stroma with spindle/stellate-shaped fibroblasts; (B) Photomicrograph (x10) showing myxoid and fibrous areas.

Histopathological examination of the surgical specimen was performed. Macroscopic examination found an irregular mass with a volume of approximately 50 cc and a solid-to-elastic consistency, appearing as a homogeneous white, slimy mass. Microscopically, it showed a loose, hypocellular tumor mass with non-atypical oval/round/spindle-pleomorphic nucleated cells embedded in a myxoid extracellular matrix with collagen fibers, consistent with odontogenic myxofibroma (Figure 3).

In the postoperative period, two weeks after surgery, an obturator was placed to close the surgical defect, thereby minimizing the ingress of food and fluids into the defect. In addition, the obturator provided functional support, improved patient comfort, and contributed to the stabilization of the remaining tissues following surgery.

The definitive diagnosis of odontogenic myxofibroma was established based on the correlation of clinical presentation, radiographic findings, and histopathological characteristics.

**Figure 4:**
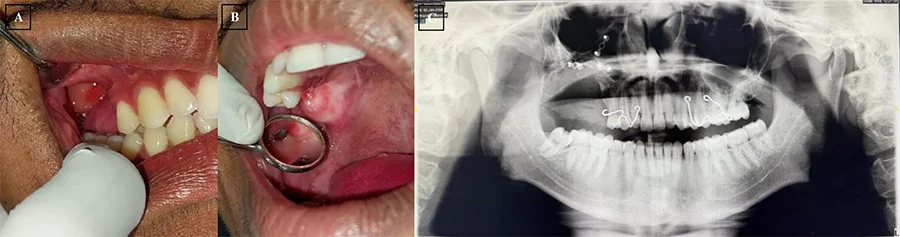
Postoperative follow-up at 16 months: (A-B) Intraoral examination; (C) Panoramic radiography.

At the 16-month postoperative follow-up, the patient remained asymptomatic with no clinical evidence of tumor recurrence. Intraoral examination demonstrated healthy mucosal coverage over the surgical site, with no signs of swelling or ulceration (Figure 4A-B). Panoramic radiographic evaluation revealed stable maxillary bone contours without evidence of recurrence, with postoperative fixation devices in situ (Figure 4C). Overall, both clinical and radiographic findings indicated satisfactory healing and absence of recurrence.

## DISCUSSION

Odontogenic myxofibroma is widely reported as a benign, painless, slow-growing tumor derived from embryonic mesenchymal tissue. Although this tumor is rare, its occurrence in the maxilla poses distinct diagnostic and therapeutic challenges due to its complex anatomy and proximity to surrounding vital structures. This case report describes a young patient with a two-year-old maxillary lesion that showed progressive enlargement and significant local extension into the maxillary sinus and nasal cavity. These features are unusual for this age group and anatomic location and can mimic more aggressive or even malignant pathology on clinical and radiographic evaluation. Consequently, this case underscores the diagnostic difficulties of odontogenic myxofibroma of the maxilla and highlights the importance of careful clinical and radiographic assessment before definitive histopathologic confirmation.

Previous literature reported that odontogenic myxofibroma most often involves the mandible and has atypical radiological features, thus potentially leading to an incorrect clinical diagnosis ^2^. However, in the present case, the tumor involved the maxillary region, which further complicated the diagnostic process. Moreover, the radiographic appearance of odontogenic myxofibroma is variable, and descriptive terms such as “soap-bubble”, “ground-glass”, and “tennis racket string” patterns have been used in the literature, which may add to diagnostic ambiguity ^6^. Therefore, based on the clinical and radiographic findings in this case, the differential diagnosis included fibro-osseous lesions, particularly ossifying fibroma and fibrous dysplasia. Distinguishing among these entities requires careful interpretation of radiographic findings combined with histopathological evaluation.

Ossifying fibroma was considered in the differential diagnosis because this benign fibro-osseous tumor may present with clinical features similar to odontogenic myxofibroma. It commonly occurs during the second to fourth decades of life and may cause facial deformity when involving the mandible or nasal obstruction when affecting the maxilla. In some cases, tooth displacement may also be observed ^(^^8^. Radiographically, ossifying fibroma demonstrates a wide spectrum of appearances, ranging from well-defined radiolucent lesions in early stages to mixed or densely radiopaque lesions as the lesion matures ^9^, which can complicate radiographic diagnosis.

Fibrous dysplasia was also considered as a differential diagnosis, as it is a slowly growing benign fibro-osseous lesion that typically presents in the first or second decade of life. When involving the maxillary region, fibrous dysplasia may extend into adjacent anatomical structures, including the nasal cavity, maxillary sinus, and orbit. On CT scan, fibrous dysplasia may exhibit ground-glass, homogenous sclerotic, or cystic patterns. Its tendency to involve surrounding structures, particularly in the maxilla, can further obscure the distinction from other benign but locally aggressive lesions ^(^^10^^,^^11^, emphasizing the potential for diagnostic confusion in the absence of histopathological assessment.

The overlapping clinical and radiographic features between odontogenic myxofibroma in the present case, ossifying fibroma, and fibrous dysplasia contributed to the diagnostic challenge in this case. As a result, histopathological examination played a crucial role in establishing the definitive diagnosis. In contrast to the fibro-osseous patterns observed in ossifying fibroma and fibrous dysplasia, histopathological evaluation of the present case revealed a hypocellular tumor composed of non-atypical oval, round, and spindle-shaped cells embedded in a myxoid extracellular matrix with collagen fibers. These findings are consistent with previously reported histopathological characteristics of odontogenic myxofibroma and enabled confirmation of the diagnosis ^(^^6^^,^^10^^,^^12^^-^^15^.

Establishing an accurate diagnosis has a significant impact on how the odontogenic myxofibroma is managed, particularly in cases involving the maxilla where clinical and radiographic features may suggest more aggressive pathology. Misdiagnosis may result in more extensive surgical resection and unnecessary additional therapy. On the other hand, inadequate tumor removal may increase the risk of recurrence. Therefore, treatment strategies, ranging from enucleation to surgical resection, should be carefully selected based on tumor size, anatomical involvement, and definitive histopathological findings ^6^.

This case highlights the diagnostic challenges associated with odontogenic myxofibroma of the maxilla, particularly due to its nonspecific clinical presentation and overlapping radiographic features with other fibro-osseous lesions. A multidisciplinary diagnostic approach integrating clinical assessment, radiographic imaging, and histopathological evaluation is essential to establish an accurate diagnosis and to guide appropriate management. Awareness of these diagnostic pitfalls may help clinicians avoid misdiagnosis and optimize treatment outcomes in similar cases.

## CONCLUSIONS

Odontogenic myxofibroma of the maxilla is a rare entity that may present significant diagnostic challenges due to its nonspecific clinical and radiographic features, which can mimic a wide spectrum of benign and malignant intraosseous lesions. This case report underscores the difficulty of establishing an accurate diagnosis based solely on clinical examination and imaging findings, highlighting the important role of histopathological evaluation in achieving a definitive diagnosis. From a clinical perspective, accurate diagnosis is essential to guide appropriate surgical management and to minimize the risk of recurrence.
